# Some Phenomena Observed in the Use of Antiseptics in Dental Surgery

**Published:** 1886-04

**Authors:** H. A. Smith


### Some Phenomena Observed In The Use of Antiseptics in Dental Surgery.

BY H. A. SMITH, D.D.S.

Read before the Mississippi Valley Association.

Of late years the study and investigation given to the large number of substances, known as "Antiseptics," because of their capability of exerting a detrimental influence on the life and growth of micro-organisms, has done much toward placing the practice of general surgery upon a scientific basis; and in our own department we have been greatly aided in developing a more exact system in the treatment of certain morbid conditions that frequently come under our notice as dentists.

Notwithstanding the study and investigations made bearing upon the relative value of the various antiseptics, there are certain phenomena presented in the treatment of special cases, that it is difficult to satisfactorily explain in the light of our present knowledge of the efficient strength of these substances. It is my purpose in this paper to call attention to some of these cases where, for some unexplainable cause, we are unable to control or counteract septic influences. For example, take a case of deep seated caries, where the pulp of the tooth is nearly encroached upon, though not in any degree exposed. In excavating such a cavity it is usual to leave a portion of decalcified or softened denture overlaying the pulp in the bottom of the cavity. This layer is treated antiseptically, with the object of destroying the micro-organisms present in the partially necrotic layer of organic matter. Following this treatment, the cavity is filled. Air and moisture are excluded, and irritation by thermal change is avoided, by judiciously selecting the filling-material. The tooth remains quiet, and to all appearances is in a healthy condition. After the lapse of a period varying from one month to several years, suddenly, and without apparent cause, a disturbance of the pulp is set up, and the inflammation passes on through the several stages; and, upon opening the pulp chamber, we find that the pulp has undergone putrefactive decomposition. Accepting the statement that the putrefactive process is always associated with mirco-organisms it is an interesting inquiry how the putrefactive organisms obtained entrance to the interior of the tooth. The cavity being sealed carefully, no infection could have been introduced from without.

We have treated the layer of carious denture covering the pulp with an agent said to be detrimental to the life and growth of the organizms undoubted by present. We have treated the cavity antiseptically; but are we sure that we have killed the organisms? Is it not probable that the agent used was not a true germicide—at least not in the dilution we have applied it, and in our treatment we have simply brought about a condition unfavorable to the growth of the organisms? This antiseptically treated layer, under the filling, is in close relation with the soft, moist tissue of the dental pulp, and in time may take up, by imbibition or otherwise moisture; or exudate from the pulp, and thus fresh nourishment is furnished to the organisms supposed to have been killed, and they begin to grow and multiply. Septic matter is developed, which, in turn, irritates the pulp. The disease progresses, and in time we find the pulp dead with the stinking products of putrefaction present, and all owing, it would appear, to our failure in rendering the softened layer of dentine covering the pulp permanently antiseptic.

But in another class of cases that occasionally come under our notice, we have putrefactive decomposition of the pulp, and yet the pulp could not have been brought in contact with germs, excepting through the general circulation. I refer to cases where the tooth has received a blow which caused the death of the pulp. After a varying length of time these teeth usually show irritation of the surrounding tissues leading to the formation of alveolar abscess. If we tap these teeth into the pulp chamber before the abscess has burst, we generally find the bad smelling products of decomposition, which indicate the presence of septic organisms. There is no breach in the continuity of the crown of the tooth, hence it becomes an interesting inquiry how the organisms came to be present in the disorganized tissue of the pulp, shut off effectually as it is from the outer world. Klein, in his late work, "*Micro-Organisms and Disease*," says, "All septic and zymogenic organisms, properly so called, differ in this essential respect from pathogenic organisms, that the former two, absolutely refuse to grow in the living tissues of a living animal." And in raising the question "Where do the micrococci and bacilli come from, which are capable of settling in a disorganized tissue, even during the life of the subject, states that the cavity of the alimentary canal, small and large intestines, especially the latter, contain, under normal conditions, innumerable masses of putrefactive micro-organisms. These being much smaller than chyle-globules, must of necessity become as easily absorbed as the latter by the lacteals, and by these are carried into the general circulation, but being putrefactive, they are unable to exist in the normal blood and normal tissues, and, therefore, in healty conditions perish. But if there be in any part a focus of disorganization, they can settle there and propagate, provided they get there through the blood in a living condition. "Many experiments," he continues, "prove that they cannot pass unscathed through the normal, healthy blood, and, therefore, it is not probable that they would reach such a focus in a living state; but let them be well enclosed in a solid particle, say of disorganized tissue, and then carried through the vascular system, and we can quite understand that in this state, i. e., in and with that particle, they may reach the distant focus in a living state, and if in this focus there is inflammation, or necrosis, we may expect them to multiply accordingly.

Having been transported to the part, the septic organisms find, in the inflamed or necrotic pulp, a suitable nidus in which to grow and set up their characteristic decomposition. If they are not thus transported, we have here a striking example of putrefaction occuring without the presence o[illegible] micrococci.

The experiments of Chaureau in performing *bistourage* of a sheep's testes before and after injecting organisms into the blood, bear some relation to the pulp cavity we have described with its dead contents. In the animals injected, the testes broke down into a putrid fluid, and violent inflammation was set up in the surrounding parts. In the others the testes underwent the series of degenerative fatty changes known as *necrobiosis*. This is the invariable course under normal conditions; and it shows, apparently, that organisms are not present normally in the sheep's testes. Again, organisms cultivated from a case of osteomyelitis, and injected into animals, caused no symptoms until their bones were injured, then osteomyelitis developed. Mere depression of the vital energy of a part, or some slight alteration of its metabolism, may, therefore, be sufficent to permit the development of a germ, which previously died in it.* But the pulps of teeth killed by a sudden blow do not, in all instances, take on these putrefactive changes. On the contrary, we sometimes find them in a shrunken or dried state, or we may find them in a moist condition, and yet remaining, as we say, "sweet."

If for any purpose we should drill into the pulp chamber of such a tooth, without observing the proper antiseptic precautions, we would most likely have irritation set up in the root membrane and parts about, caused by micro-organisms being admitted with air and the fluids of the mouth, through the artificial opening. Usually we treat such a root with some approved antiseptic, until all symptoms of inflammation have subsided. The canal is filled with a fibrous material well saturated with our favorite antiseptic. The opening is carefully stopped, so that no air or moisture can be admitted. The tooth remains quiet for a month, it may be a year, when suddenly it becomes tender to the touch, threatening abscess. We unstop the root, and find the contents offensive, denoting the presence of septic germs. They could not have entered from the outside. Have they been transported in the manner referred to? Is it not more probable, since we know that organisms were once in the root canal, that our supposed germicide has not annihilated the life of the micro-organisms, as we expected, and that in its use we have simply brought about a condition that, for an uncertain period, has had only the effect to retard the growth of the organisms in question?

I stated in the beginning that the study of the properties of

*Green's Pathology, p. 434.

antiseptics had done much to aid us in the intelligent treatment of morbid processes frequently observed in dental surgery. We need only contrast the results obtained under the old system, with those had by the strict observance of the most approved antiseptic methods, to verify the truth of this.

And yet we are quite in the dark as to whether the substance that we call an antiseptic is, in the true sense, a germicide ; that is, having the power of annihilating the life of the particular organism with which we have to deal. The phenomena observed following the most intelligent use of antiseptics in the oral cavity, to which your attention has been called, show that there is before us an interesting field for research.

In dental practice we have frequent opportunities of testing the strength of antiseptics that seldom offer in general surgery. The pulp chamber may be made a closed cavity, and these agents may be used in the highest dilution without much danger of bad results. We should, therefore, seize upon the favorable opportunities open to us, and institute a rigid and systematic series of investigations as to the comparative value of the numerous substances that are now brought to our notice as antiseptics.

